# Secular trends in mental health problems among young people in Norway: a review and meta-analysis

**DOI:** 10.1007/s00787-024-02371-4

**Published:** 2024-02-16

**Authors:** Thomas Potrebny, Sondre Aasen Nilsen, Anders Bakken, Tilmann von Soest, Kirsti Kvaløy, Oddrun Samdal, Børge Sivertsen, Heidi Aase, Lasse Bang

**Affiliations:** 1 Department of Health and Functioning, Western Norway University of Applied Sciences, Bergen, Norway; 2 Regional Centre for Child and Youth Mental Health and Child Welfare, NORCE Norwegian Research Centre, Bergen, Norway; 3 Norwegian Social Research (NOVA), Oslo Metropolitan University, Oslo, Norway; 4 Department of Psychology, PROMENTA Research Center, University of Oslo, Oslo, Norway; 5 Department of Public Health and Nursing, Faculty of Medicine and Health Sciences, HUNT Research Centre, Norwegian University of Science and Technology NTNU, Trondheim, Norway; 6 Department of Community Medicine, Faculty of Health Sciences, Centre for Sami Health Research, UiT The Arctic University of Norway, Tromsø, Norway; 7 Levanger Hospital, Nord-Trøndelag Hospital Trust, Levanger, Norway; 8 Department of Health Promotion and Development, University of Bergen, Bergen, Norway; 9 Department of Health Promotion, Norwegian Institute of Public Health, Bergen, Norway; 10 Department of Research and Innovation, Helse-Fonna HF, Haugesund, Norway; 11 Department of Child Health and Development, Norwegian Institute of Public Health, Oslo, Norway

**Keywords:** Mental health problems, Trends, Youth, Review, Meta-analysis

## Abstract

There is a growing concern that the mental health of recent generations of youth is deteriorating, yet the etiology of these secular changes is not fully understood. We aimed to review the evidence on trends in mental health problems among young people in Norway. Seven large-scale repeated cross-sectional studies were included in this study, comprising 35 cross-sectional data collections between 1992 and 2019, with a total sample of 776,606 young people. Our study found a clear increase in mental health problems among young females in Norway over the past few decades, while the trends were less marked for males. The proportion of individuals scoring above the problematic symptom score threshold increased on average by 11.2% (range 2.2% to 21.9%) for females and 5.2% (range − 0.9% to 11.1%) for males, based on data from the individual studies. The results from a meta-regression analysis showed that across all surveys, mean symptom scores increased by 17% (95% CI 12 to 21%) among females and 5% (95% CI 1 to 9%) among males from 1992 to 2019. Overall, mental health problems have increased continually since the early 1990s among young people, especially among young females. The cause of these secular changes remains unknown but likely reflect the interplay of several factors at the individual and societal level.

Protocol registration: Open science framework, November 8, 2021 (https://osf.io/g7w3v).

**Supplementary Information:**

The online version contains supplementary material available at 10.1007/s00787-024-02371-4.

## Introduction

Studies have shown an increase in self-reported mental health problems among young people (13–24 years of age) in high-income countries over the past few decades, particularly among females [1–4]. The increase in mental health problems among the youth also coincides with other indirect indicators of mental health problems, such as increasing rates of mental healthcare utilization and treatment [2, 5]. Clinicians, researchers, and policymakers have raised concerns about whether today’s youth may be more prone to mental health problems than previous generations on a global scale.

A comparative systematic review and a large comparative study, comparing 36 countries across various geographical regions, highlight a noticeable increase in mental health problems among young people from 1980 to 2018. This increase appears more pronounced in countries located within Northern Europe [3, 6] and possibly Western Europe [6] compared to other regions. The authors suggest that conducting country-specific analysis of trends in mental health problems is needed to further understand the nature and underlying causes of these secular trends [3, 6].

Within Northern Europe, Norway is highlighted as one of the countries with the largest increase in mental health problems among young people over time [7]. In Norway, numerous studies have tracked self-reported mental health problems among Norwegian youth over several decades, using a repeated cross-sectional design, which is ideal when studying societal and population-level changes over time [8]. These studies are generally population-based, have well-defined sampling frames and cover the period from 1992 to the present. They all use a self-report symptom checklist to assess mental health problems, primarily focusing on internalizing problems, such as symptoms of anxiety and depression. Individually, these repeated cross-sectional surveys all indicate that self-reported, mental health problems among young Norwegians have increased at various time periods during the past 40 years [9–14]. This is particularly concerning when considering trends in light of medical and other population data that show a parallel increase in both the use of healthcare services, the diagnosis of mental disorders, the use of antidepressants, and surging self-harm rates among Norwegian youth [15–17]. Many individual surveys also indicate that these trends are more pronounced among young females, while findings for young males are more ambiguous [9–14]. There is, however, substantial variation between individual surveys concerning estimates of point prevalence, symptom score variations, and the magnitude and steepness of the increase over time [3]. This variation makes it difficult to draw strong inferences about how much young people’s mental health has actually changed in Norway and the potential policy implications that this may have.

One way forward, to obtain a clearer understanding of the secular trends in mental health problems among young people in Norway, is to synthesize the findings across all repeated cross-sectional surveys. Since secular changes in mental health problems have considerable implications for public health, education, healthcare services, treatment, and policymaking, it is imperative that these secular changes are better documented and understood. The COVID-19 pandemic has certainly underscored the importance of understanding the general trends in mental health problems prior to the pandemic, to differentiate these from changes relating to the pandemic itself [18, 19]. Summarizing the numerous high-quality surveys that have been repeated over 3 decades in Norway will provide a unique opportunity for better documentation and will help understand the secular trends prior to the COVID-19 pandemic.

In the current study, we investigated the secular trends in mental health problems among young people in Norway by aggregating and synthesizing data from all repeated cross-sectional surveys. We present the findings from each individual survey, as well as a meta-analysis to provide pooled estimates of the secular changes of mental health problems.

## Methods

### Search strategy and inclusion criteria

This review was structured in accordance with the Preferred Reporting Items for Systematic Reviews and Meta-Analyses (PRISMA) guidelines [20]. A study protocol was published in the Open Science Framework (OSF) in advance of the study (https://osf.io/g7w3v).

All Norwegian repeated cross-sectional surveys were eligible for inclusion, provided that they assessed general mental health problems (i.e., symptoms of anxiety and depression) among 13–24 years old and made attempts to attain a representative sample of the general youth population. Each survey had to include data collected on at least two occasions using similar recruitment and outcome measures. Since this is a study relating to mental health problems among the youth population, in general, surveys from clinical- or at-risk samples were not included.

### Data collection process

The studies eligible for inclusion in this review were known in advance by Norwegian youth health researchers and experts. The principal investigators from all Norwegian youth surveys were invited to collaborate in advance on this study. The study data were drawn from open repositories or via direct contact with the principal investigators of each youth survey in 2022.

A major advantage of this approach was that it allowed us to request information regarding relevant outcomes or indicators directly from the individual youth survey administrators, information which is not widely available or reported on previously.

Data extraction and verification was done by three authors (TP, SAN, and LB).

### Operationalization of mental health problems in the included studies

After identifying studies based on our a priori inclusion criteria, there were two different operationalizations of mental health problems in the studies, both of which were self-report symptom checklists: The Hopkins Symptom Checklist (HSCL) and the Health Behaviour in School-aged Children Symptom Checklist (HBSC-SCL). Here, we will briefly present these instruments.

#### The Hopkins Symptom Checklist (HSCL)

The HSCL was developed as a broad measure of mental health problems, defined by the frequency of symptoms of mental health problems in clinical and non-clinical samples. The instrument originally consisted of 90 items [21]. However, shorter formats (5–25 items) have since been developed, focusing mainly on symptoms of anxiety and depression. These short versions have been comparable with the longer versions and perform well (the correlation between the different versions of the HSCL ranges from 0.91 to 0.97) [22]. The respondents were asked to what extent the symptoms have bothered them over the past 7 or 14 days. The sample items were “feeling hopeless about the future”, “feeling everything is an effort”, “suddenly scared for no reason”, and “feeling tense or keyed up”. The responses were recorded on a 1–4 scale, ranging from “not at all” to “a little” to “quite a lot” to “extremely”, with higher scores signifying more severe mental health problems. The responses are typically either averaged to produce a total HSCL score or dichotomized based on recommended cut-off threshold (for the shorter formats) of either 2.0 [22] or 3.0 [23, 24]. Previous studies have shown that young people scoring above this threshold were within the range of depressive disorder [22–24].

#### Health Behaviour in School-aged Children Symptom Checklist (HBSC-SCL)

The HBSC-SCL was designed to assess mental health problems according to the frequency of symptoms in non-clinical youth samples. The HBSC-SCL measures eight symptoms: headache, abdominal pain, backache, feeling low, irritability or a bad mood, feeling nervous, experiencing sleeping difficulties, and dizziness. Young people were asked how often they had experienced these symptoms over the past 6 months. The responses were recorded on a 1–5 scale, namely, “about every day”, “more than once a week”, “about every week”, “about every month”, and “rarely or never”. Greater symptom frequency indicated more significant mental health problems [25]. The responses are recommended to be averaged to a total HBSC-SCL score and there is no clear agreement on a cut-off threshold [25]. Previous research supports the validity and reliability of the instrument [19, 20, 26, 27].

### Data analysis

#### Individual survey analyses

We extracted key variables from each survey in a harmonized manner. This included the mean symptom scores for each individual participant and their standard deviations, stratified by survey year, sex, and age.

To examine the secular changes of mental health problems within each survey, we fitted a series of linear and logistic regression models. First, we performed linear regression analyses to investigate the secular change in the mean symptom scores for each survey separately. To provide a standardized measure of effect size, we z-transformed (set the grand mean equal to zero and a standard deviation equal to one) the symptom scores within each survey separately for males and females. In these models, the mean symptom scores (dependent variable) were regressed on survey year and age (independent variables). The survey year was dummy coded using a backward difference contrast coding scheme, whereby each survey year was compared to the prior level (i.e., 2002 vs. 1998; 2005 vs. 2002). This generated *n* − 1 contrasts, where *n* is the total number of survey years. All models were fitted separately for males and females.

Second, we performed binary logistic regression analysis to investigate the secular change in proportions of individuals scoring above a problematic symptom score threshold. We defined the problematic symptom score by employing a cut-off of a mean score  ≥ 2. This is a threshold commonly used in many Norwegian youth surveys to indicate high symptom load and is the suggested threshold for identifying a mental disorder in the shortest versions of the Hopkins Symptom Checklist-5 (HSCL-5) [22]. The dependent variable in these models was a binary variable, denoting whether an individual scored above or below the cut-off. The independent variables were survey year and age, and the models were run separately for males and females.

For each survey, we plotted the mean symptom scores and proportions of individuals scoring above the cut-off threshold of  ≥ 2 across time. As a cut-off threshold of  ≥ 3 is also commonly used when reporting findings in some of the included surveys, we plotted the proportions of individuals scoring  ≥ 3 across time for descriptive purposes. For each model, we reported the associations between the dependent variable, and survey year and age. The SPSS software [28] was used for these analyses and alpha was set to 0.05 for all analyses.

#### Meta-analysis

For each individual survey, we collected means and standard deviations of the outcome measures. These estimates were then pooled and synthesized using a multilevel meta-regression analysis as the primary meta-analytic technique, by utilizing the package, “Metafor”, in the R statistical environment [29, 30]. As the mean symptom scores from different surveys varied on a relative scale, the mean symptom scores were log-transformed to express the outcome score on a comparable metric, indicating relative change on the log scale. This procedure “normalizes” the relative differences between the surveys, thereby rendering the differences between the surveys interpretable and ensuring the validity of inference [31].

The data had a natural multilevel structure with three levels; outcomes from individual participants from the primary surveys (level 1), outcomes summarized from individual surveys at each data collection (level 2), and outcomes clustered within each survey (level 3). We expected that the outcome within the surveys would be fairly homogeneous, but with substantial variance between surveys, due to the differences in the instruments used, the sampling procedure, and the time period of the data collection. To model and correct for this complex three-level structure, we modeled a three-level meta-regression, examining within-cluster heterogeneity at level 2 (the variation of the true effect size within studies) and between-cluster heterogeneity at level 3 (variation between surveys) using restricted maximum-likelihood estimation. The *I*^2^ statistic was computed as an indicator of heterogeneity in percentages, with values 0–50% indicating no heterogeneity, 50–75% indicating moderate heterogeneity, and 75–100% indicating substantial heterogeneity [32].

The amount of (residual) heterogeneity accounted for in the full three-level model can be regarded as a (pseudo) *R*^2^ value (corresponding to the interpretation of a traditional adjusted *R*^2^ value) and is the percentage of the variance explained [33]. The full model (three levels) was compared to a reduced model (two levels) to assess model fit using the likelihood ratio test.

To inform our modeling strategy, we conducted preliminary analyses comparing the fit of a linear and non-linear functions (quadric and cubic functions) of the time trend. Both on the total sample and gender stratified samples, a linear function of the time trend had best fit to data and was therefore chosen in our substantive analyses (see Supplementary Table S3 and Supplementary Figure S1).

The final three-level meta-regression results are presented as the relative change in the (log-) mean symptom scores (95% CI) per year, including differences by sex, and the interaction effect between sex and year. In addition, regression estimates are adjusted for age and centered at the mean age across the samples (age 17). Alpha was set to 0.05 for all analyses.

## Results

### Characteristics of included studies

Our review included seven repeated cross-sectional surveys, investigating mental health problems among young Norwegian people:

1. Young in Norway (YiN).
2. Young in Oslo (YiO).
3. The Trøndelag Health Study (Young-HUNT).
4. Students’ Health and Wellbeing Study (SHoT).
5. Living Conditions Survey (SILC).
6. Health Behaviour in School-aged Children (HBSC).
7. The Ungdata Survey.

These seven surveys comprise 35 separate cross-sectional data collections carried out between 1992 and 2019 and contain data on self-reported mental health problems among *n* = 776.606 young people between the ages of 13 and 24. Sex was generally evenly distributed across the surveys, with females comprising 47.4% to 69.2% of the survey samples. See Table 1 for the key characteristics of each survey dataset, as included in the current study.

**Table 1:** Overview of included repeated cross-sectional surveys

Survey	Year(s)	Sample *n*	Female (%)	Age *M* ± *SD*	Outcome^a^	Outcome range	Population	Response rate	Response level
YiN	1992	10,612	50.8%	15.9 ± 2.09	HSCL-6	1–4	National	97.0%	Individual
	2002	11,149	51.4%	15.7 ± 1.8	HSCL-6	1–4	National	91.0%	
	2010	7343	50.6%	14.9 ± 1.8	HSCL-6	1–4	National	84.3%	
Young-HUNT	1995–1997	8653	49.8%	16.1 ± 1.8	HSCL-5	1–4	Trøndelag County	88.1%	Individual
	2006–2008	7911	50.8%	15.9 ± 1.7	HSCL-5	1–4	Trøndelag County	78.4%	
	2017–2019	7603	51.4%	16.1 ± 1.8	HSCL-10	1–4	Trøndelag County	76.0%	
YiO	1996	10,822	49.6%	15.4 ± 0.9	HSCL-6	1–4	Oslo county	94.0%	Individual
	2006	10,856	52.0%	15.3 ± 0.9	HSCL-6	1–4	Oslo county	92.7%	
HBSC	1994	3334	48.9%	14.5 ± 1.1	HBSC-SCL	1–5	National	82%	Individual
	1998	3267	49.2%	14.5 ± 1.1	HBSC-SCL	1–5	National	93%	
	2002	3343	50.3%	14.4 ± 1.0	HBSC-SCL	1–5	National	88%	
	2006	3096	47.4%	14.5 ± 1.1	HBSC-SCL	1–5	National	85%	
	2010	2646	49.3%	14.6 ± 1.0	HBSC-SCL	1–5	National	81%	
	2014	1930	52.1%	14.5 ± 1.1	HBSC-SCL	1–5	National	76%	
	2018	1456	51.1%	14.5 ± 1.1	HBSC-SCL	1–5	National	84%	
SILC	1998	877	53.6%	19.7 ± 2.7	HSCL-25	1–4	National	79.2%	Individual
	2002	611	52.5%	19.9 ± 2.7	HSCL-25	1–4	National	71.9%	
	2005	591	54.7%	19.6 ± 2.6	HSCL-25	1–4	National	73.1%	
	2008	461	56.6%	19.4 ± 2.7	HSCL-25	1–4	National	66.1%	
	2012	436	57.1%	19.8 ± 2.6	HSCL-25	1–4	National	57.4%	
	2015	1183	49.0%	19.8 ± 2.6	HSCL-6	1–4	National	61.7%	
	2019	1074	47.0%	20.1 ± 2.6	HSCL-6	1–4	National	54.8%	
SHoT	2010	5962	65.8%	23.1 ± 3.3	HSCL-25	1–4	National	23.5%	Individual
	2014	13,525	66.5%	23.8 ± 3.3	HSCL-25	1–4	National	28.5%	
	2018	49,730	69.2%	23.2 ± 3.3	HSCL-25	1–4	National	31.5%	
Ungdata	2010	17,725	49.5%	14.4 ± 1.2	HSCL-6	1–4	National	80.3%	Individual
	2011	11,971	51.0%	14.5 ± 1.2	HSCL-6	1–4	National	82.3%	
	2012	24,079	49.4%	14.7 ± 1.2	HSCL-6	1–4	National	85.2%	
	2013	81,541	49.7%	14.7 ± 1.4	HSCL-6	1–4	National	> 80%	
	2014	45,525	50.6%	14.9 ± 1.5	HSCL-6	1–4	National		
	2015	70,81	49.9%	15.1 ± 1.6	HSCL-6	1–4	National		
	2016	68,007	50.5%	14.9 ± 1.4	HSCL-6	1–4	National		
	2017	100,361	50.4%	15.1 ± 1.6	HSCL-6	1–4	National		
	2018	70,579	49.6%	15.2 ± 1.6	HSCL-6	1–4	National		
	2019	117,567	50.4%	15.3 ± 1.6	HSCL-6	1–4	National		

^a^*HSCL* Hopkins Symptom Checklist, *HBSC-SCL* Health Behaviour in School-aged Children

### Analysis of individual youth surveys

Most of the surveys are nationally representative, except for the SHoT which represents the overall student population. Subsequently, the sex distribution in the SHoT samples can be slightly skewed, due to the fact that females outnumber males in the university setting. Furthermore, most surveys use a cluster-randomized sampling procedure, based on lists of schools or school classes; a notable exception is Ungdata, which is a census used for municipal health impact assessments and planning. All but one survey used a variation of the HSCL to assess mental health problems [21]. One repeated cross-sectional survey used the HBSC-SCL [25]. All but one survey used the same symptom measure across time. The exception was the SILC survey, which used the HSCL 25-item version for the first five collections (1998 to 2012), then changed to a 13-item version for the sixth collection (in 2015), and finally changed to a six-item version for the seventh collection (in 2019) (Table 1). An additional note is that the specific items included in the various HSCL versions across studies were not necessarily the same.

We identified two additional surveys that were eligible for inclusion in our study (the Hedmark Youth Survey and the Østfold Youth Survey). These surveys are older, smaller in terms of scale, are regionally designed and are no longer in operation. Despite our efforts, we were unable to gain access to the survey data or obtain a comparable summary effect from these studies; therefore, they were not included in the current study. We note, however, that the results from these studies are in line with the findings presented later in this review, based on white paper documents [34, 35].

#### Trends in mental health symptoms scores among young people by study

**Fig. 1:**
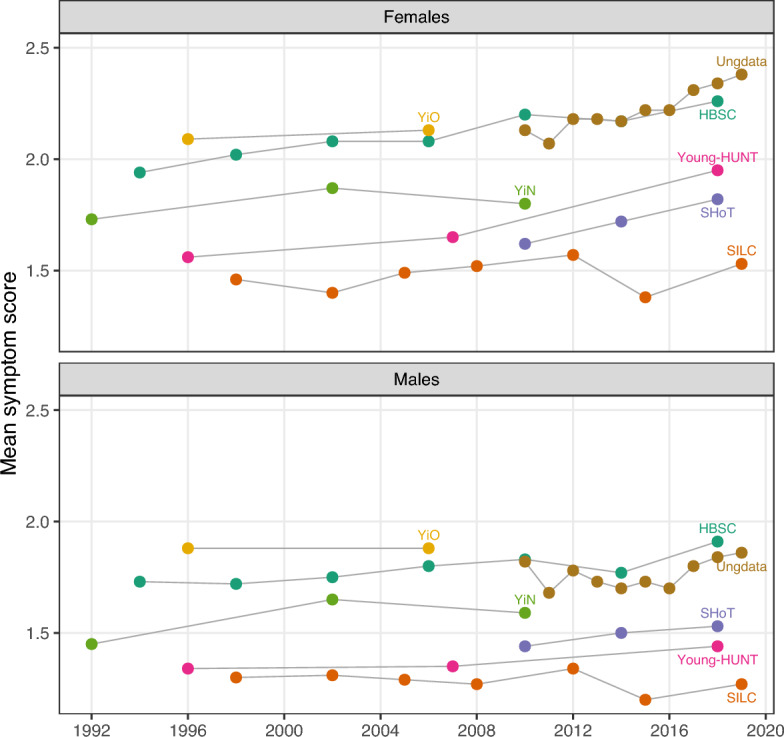
Mean symptom scores across time, stratified by sex and survey

Based on the individual survey results, all repeated cross-sectional surveys showed an increase in mean symptom scores from the first to the last survey year, particularly among females (see Fig. 1). Results from the linear regression models confirmed that all surveys pointed to significant increases in mean symptom scores at some time point in time, for both males and females. In relation to females, no surveys indicated significant declines in mean symptom scores at any point, compared to the baseline. Among males, surveys show a similar increase over time overall, but results were generally more mixed (Supplementary information; Table S1).

#### Trends in problematic mental health symptom scores among young people by study

**Fig. 2:**
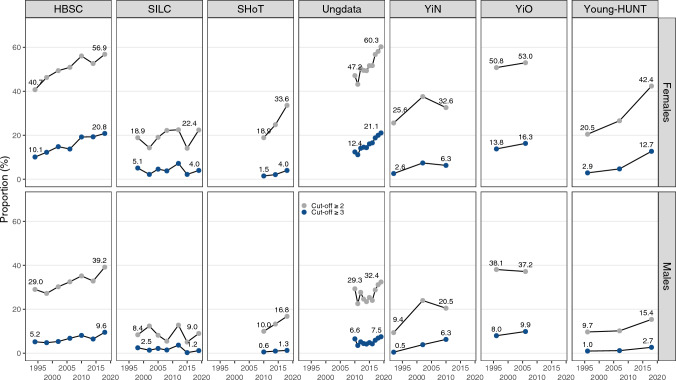
Proportions scoring  ≥ 2 and  ≥ 3 in the symptom outcome measures by survey, survey year, and sex

The proportions of individuals scoring above the problematic score threshold(s) generally increased from the first to the last survey year, similar to that of mean symptom scores. This was especially evident among females (see Fig. 2). The proportion of individuals scoring above the problematic score threshold (≥ 2) increased, on average, by 11.2% (range 2.2% to 21.9%) for females and 5.2% (− 0.9% to 11.1%) for males from the first to the last survey year. The largest change was observed among young females in the Young-HUNT Study, in which the proportion scoring above the problematic score threshold (≥ 2) increased from 20.5% to 42.4% between 1995 and 2019. In all surveys, a higher proportion of females than males scored above both cut-offs across all survey years. There was, however, considerable variability between surveys (Fig. 2). The results from the binary logistic regression models suggest an increase in the proportion scoring above the problematic score threshold at certain time points for both males and females (Supplementary information; Table S2). No surveys showed significant decreases for females overall, compared to the baseline. Among males, the results were again generally more mixed.

Furthermore, results of the individual surveys suggest that there was a positive association between age and mental health problems. Older youth were more likely to have a higher mean symptom score or score above the problematic score threshold across the individual surveys. The only exception was for females in the SHoT sample where age was negatively associated with symptom scores and problematic scores. The sample was slightly older, which likely account for the conflicting results (Supplementary information; Table S1 and Table S2).

### Evidence of increasing mental health problems among young people

**Fig. 3:**
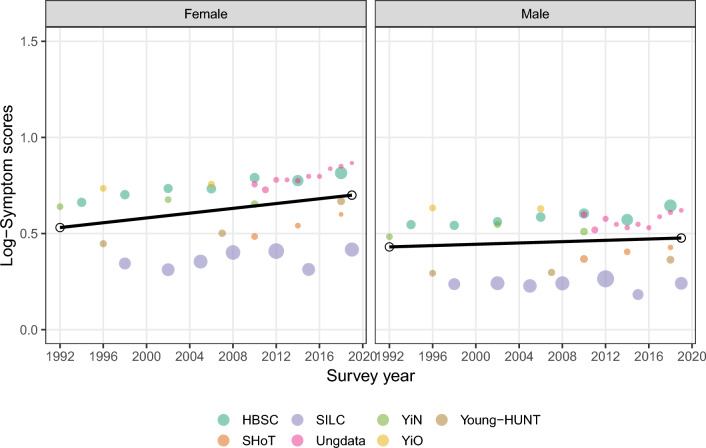
Estimated trends in the scores of mental health problems among young people by sex against the unadjusted values on a log scale (larger circles indicate greater variance)

Results from the pooled three-level meta-analytic model indicated an increase in the log-mean score of mental health problems among young people in Norway between 1992 and 2019. The interaction effect between sex and year was significant, suggesting a greater increase in symptom scores across the years for females compared to males. More specifically, as the results from Table 2 show, the mean symptom scores increased annually from the year 1992 onwards by approximately 0.2% for males and 0.6% for females. In 2019, 27 years later, the annual increase accumulated to a relative increase in mean symptom scores of 5% for males (95% CI 1 to 9%) and 17% for females (95% CI 12 to 21%), adjusted for age- and between-survey differences. Moreover, the significant effect of sex indicated that females generally had more mental health problems at any given time point (a higher symptom score of approximately 10%). Figure 3 shows the estimated symptom score increase, accounting for within- and between-survey heterogeneity and age differences against unadjusted symptom scores, on a log scale.

**Table 2:** Adjusted multilevel meta-regression estimates of annual change in mental health problems among young people between 1992 and 2019

Predictors	Log-symptom scores	95% CI	*p*
Intercept	0.430	0.247–0.614	< 0.0001
Year	0.002	0.001–0.003	0.048
Sex (female)	0.101	0.062–0.140	< 0.0001
Year: sex (female)	0.005	0.002–0.007	< 0.0001

In the context of trying to understand and quantify the sources of variation in youth health data, we compared different statistical models trying to account for variation both within individual surveys and differences between surveys. We found that a three-level model provided a significantly better fit compared to a two-level model, with level 3 heterogeneity constrained to zero (*χ*^2^_1_ = 112.47; *p* < 0.001). In the case of the three-level model, the estimated variance component was *τ*^2^_Level 3_ = 0.06 and *τ*^2^_Level 2_ = 0.01. This suggests that 98.13% (*I*^2^_Level 3_) of the total variation can be attributed to between-survey variation (high between-survey heterogeneity in the outcome) and 1.85% (*I*^2^_Level 2_) to within-survey heterogeneity (no substantial heterogeneity within individual surveys). The *R*^2^ coefficient indicated that the three-level meta-regression model reduced 89.8% of the initial heterogeneity variance compared to a model not accounting for the multilevel structure of the data [36]. This suggests that considering between-survey differences is crucial for explaining and reducing variation when conducting a meta-analysis on youth health data in a country like Norway.

## Discussion

### Overview of main findings

This review and meta-analysis aimed to provide an overview of the secular trends in mental health problems among young people in Norway, by synthesizing data from all large-scale, repeated cross-sectional surveys. Seven surveys were included, covering 35 measurement points between 1992 and 2019, with a total sample of 776,606 young people. Our study demonstrates an increase in self-reported mental health problems among young females in Norway over the past few decades, while trends are less marked for males. In the individual surveys, the difference in the proportion of individuals scoring above the problematic score threshold from the first to the last survey year was, on average, 11.2% for females and 5.2% for males. Pooled meta-regression estimates showed that from 1992 to 2019, the mean symptom scores increased by approximately 17% for females and 5% for males.

### A comparative perspective on the trends in mental health problems among young people

Our findings align with prior studies from other high-income countries in Europe [3, 37–43] and North America [44, 45]. Combined, these studies show that self-reported mental health problems among young people have increased over the past 3 decades, particularly among females. The findings for males are more mixed, however, with certain studies identifying increases over time [39, 42, 44] and others finding more stable or decreasing trends [37, 38, 41]. This was also the case in our study, as we pinpointed shorter periods indicating flat or even declining trends in some surveys (e.g., see [13]). However, over a longer time period, the studies all show a general increase over time and secular trends in self-reported mental health problems coincide with increasing rates of treatment for mental disorders among young people [5, 46–49].

Other studies from other country contexts have noted trends of mental health problems among youth may depend on survey characteristics like sex, age, study period, country, and outcome measures [1–3, 43]. For example, a review of 36 UK surveys of children and youth found large variations both within and between surveys based on survey characteristics, such as country, time, age, and outcome measure [43]. The researchers observed an increase in long-standing mental health conditions but a stable trend for measures of psychological distress and emotional well-being. In line with Pitchforth and colleagues [43], our findings also indicated substantial variation between comparable national surveys, which could significantly affect precision if not appropriately addressed. However, even after accounting for between-survey characteristics and comparing our findings with other mental health outcomes, such as reports of increase of diagnoses of mental health disorders, increased use of healthcare services, increased use of antidepressants, and increasing rates of self-harm among Norwegian youth [15–17], the evidence consistently shows deteriorating mental health among young people in Norway, particularly among young females, between the 1990s and the present.

Other extensive comparative studies have also emphasized the need for caution when comparing cross-national trends, given the substantial variation within and between nations, even when employing similar mental health outcomes [3, 6]. Country-specific societal factors, such as policy or economy difference, have the potential to strongly influence or mediate the trajectories of mental health problems among youth populations, thus leading to genuine cross-national differences [50]. However, as our findings show, in line with Pitchforth and colleagues [43], survey characteristics substantially influence outcome variation even within nations. This underscores the need for consistent methodology, identifying the best methodological practices, and the use of a common instruments to enhance precision in country-specific estimates of mental health problems. Additionally, improving methodological consistency within countries can feasibly enhance validity when comparing mental health problems cross-nationally in the future.

### Has there been a general increase in mental health problems among young people?

The secular trends identified in the previous studies and in our own are exclusively related to internalizing problems as opposed to externalizing problems. Studies that have assessed both externalizing and internalizing problems suggest that only the latter have increased over time, among young people [38, 40, 51]. Therefore, the observed increase in mental health problems appears to be specific to symptoms of anxiety and depression (e.g., see [37]). This is also mirrored in the rates of treatment for mental disorders, where the proportions treated for externalizing problems have decreased over time [17, 47]. Unfortunately, the surveys included in our review did not include standardized and comparable measures of externalizing problems. However, there are no indications otherwise that externalizing problems have increased in Norway the past decades—if anything available evidence (statistics produced publicly by Norwegian health registries) suggests that such problems have decreased in the youth population [17]. Furthermore, the increase in mental health problems is related to age and more pronounced among older youth, compared to child and adult populations [10–12, 39, 44]. Studies of children under the age of 11 generally show no increasing mental health problems over time [1, 2, 11, 52]. None of the surveys included in our review included children under the age of 13 years, so we were unable to investigate trends in this age group further. A recent Norwegian population study found that mental health problems increase over the last decades among young people, but not for adults [10]. In fact, declining rates of mental health problems were evident among those aged 60 or older. This may suggest that increasing mental health problems are specific to internalizing problems among cohorts of female youth, as opposed to constituting a broader phenomenon.

### Potential causes of the trends in mental health problems

Several explanations for the increasing trends in mental health problems both in Norway and internationally have been proposed; changes in health-related behaviours, frequent social media use, increasing school-related stress, and greater willingness to report symptoms of ill-health.

To our knowledge, only two publications, which utilize the included survey data, have empirically investigated a range of potential causes for the highlighted Norwegian trends in our review. One of these [13] suggests that the increase in self-reported depressive symptoms among boys and girls from 1992 to 2002 could be partially attributed to increases in eating problems and cannabis use. Reduced satisfaction with own appearance also appeared to contribute, particularly among girls. The second study [14] found that increase of self-reported eating problems, such as bulimia nervosa symptoms and food preoccupation, over time could be partially linked to appearance satisfaction, alcohol intoxication and global self-worth. However, these studies generally found that these proposed mechanisms only account for a small part of the increasing trajectories of mental health problems between 1992 and 2010. Additionally, other mechanisms might underlie the continued increase in mental health problems after 2010.

In contemporary discussion around deteriorating mental health among young people, the “social media hypothesis” is garnering the most attention. The social media hypothesis posits that excessive social media use might be the major cause of the surge of mental health problems among youth, after around year 2007 [53]. Recent reviews do indeed suggest that there might be a weak association between social media and mental health problems [54–56]. However, there is a lack of evidence to establish whether this association is causal [57]. An important prior study leveraged a natural experimental design and reported that the introduction and expansion of social media, specifically Facebook, in American student communities in 2004–2006 had an adverse impact on their mental health [58]. However, this study used data collected during the emergence of social media and there is a lack of similar studies that capture the past decade’s developments in social media platforms. Additionally, there is some evidence that the negative effects of social media use are more pronounced for females than males [59, 60]. This seemingly fits the pattern that mental health problems have increased more among females than males. Nevertheless, the effect of social media use on mental health remains a subject of the ongoing debate and there is a need for future research that can establish the degree to which social media use can account for the increasing trend of mental health problems observed in Norway and other high-income countries.

In light of the current study, social media also cannot account for the observed increase in mental health problems that occurred before the huge expansion of social media platforms. The decades prior to 2007 were also characterized by increasing screen time in relation to e-mailing, Internet browsing, and computer gaming. Such activities have been associated with poor perceived health (mediated through negatively affecting sleeping habits) [61] and negative physical complaints [62]. It is important to account for the increases in mental health problems prior to the emergence of social media as well, as several studies including our own, indicate that the deterioration of mental health among young people was evident even before the emergence of social media (e.g., [63]).

Others have suggested that school-related stress and pressure have contributed to the mental health trajectories [64]. This has been spurred by observations that stress and pressure related to school have increased over the past decades [6, 42, 65]. Large comparative studies of 43 countries in Europe and North America demonstrated an increase in schoolwork pressure and its association with increased mental health problems over time [6, 65]. However, these associations were generally modest. Another study [66] found that the effects of school stress on psychosomatic symptoms became stronger over time in the period between 1993 and 2017, but that school stress only partly explained the increase in such symptoms. Interestingly, one study showed that the association between school stress and mental health problems was stronger for countries that were richer and more educated, suggesting that societal factors may influence trends of mental health problems [50]. The aforementioned studies focus on a relatively recent time period (i.e., from 2000 and onwards). A less recent study conducted in Scotland [67] showed that school disengagement and worries about school were among the explanatory factors most strongly associated with the increase in mental health problems in the time period from 1987 to 2006. This suggests that the association between school-related variables and mental health trajectories may have persisted over the last 3 decades. Despite the evidence that support the notion that school-related stress and pressure may have contributed to the rising trajectories of mental health problems, such contributors only account for a relatively small portion of the trends.

It has also been suggested that the increasing trend of mental health problems could be “inflated”, due to reduced societal stigma and a subsequent increased willingness to report symptoms [2]. If willingness to report mental health symptoms had changed over time, one would expect to find signs of factorial invariance across time, when assessing the psychometric properties of the instruments used, which at present, does not seem to be the case [13, 68]. Moreover, a study conducted in the UK found that improvements in attitudes toward mental illness did not mirror changes in self-reported mental health problems across English regions over the past decade [69]. In addition, evidence from experimental studies suggests that training youth to recognize symptoms of mental health problems does not influence mental health problem outcomes, at least in a controlled setting [70].

While conclusive causal analyses regarding deteriorating mental health in Norway are lacking, several concurrent societal trends might have contributed to the observed increase in mental health problems. Recent national studies indicate that young people are dedicating more time to digital screens and social media, and subsequently spend less time with friends [9]. Additionally, negative attitudes toward school have increased during the past decade [9]. Moreover, there has been a notable reduction in the stigma surrounding mental health problems in recent decades, encouraging today’s youth to be more open about such issues compared to older generations.

Despite increased efforts to understand the determinants of increasing mental health problems, there is still a need for future research to extensively examine the potential causes. Considerable challenges persist in establishing causality between explanatory factors and secular trends in mental health problems. Where associations do exist, they are generally small and feasibly account for only a small portion of the total increase in mental health problems over time. It seems more and more unlikely that there is a single catalyst for increasing mental health problems among young people, but rather that several determinants working together to drive the negative trend in Norway and several other high-income countries.

### Limitations

A major strength of this study is that it includes a sample of over 770,000 to examine the trends in youth mental health problems in Norway, which provides a solid evidence base for public health decision-makers. However, several limitations should be noted. First, based on the protocol for this study, we planned to include socioeconomic status and minority background in our analyses. Unfortunately, we were not able to include comparable measures of socioeconomic status and minority background as explanatory variables in our data extraction and analysis, due to the data being missing or not comparable across surveys.

Another limitation is that despite the individual surveys included in this study having both comparable designs, and being drawn from the same population, there were still substantial variations between them. Other factors not directly examined in this study, that might have contributed to between-survey differences could be (a) the difference in the response rate between surveys and the change in rates over time, (b) the sampling procedure and efforts to control for low initial response rates, (c) and the length and content of the different survey questionnaires (e.g., various versions of the main outcome measure). This does, however, emphasize the need for a common instrument and consistent methodology when operationalizing mental health problems. Further research into psychometric properties, item functioning, and other validation work on youth health measures can provide further insights into the current youth trends.

## Conclusion

In conclusion, mental health problems have been increasing continually since the early 1990s among young people in Norway, especially among young females. The causes of these secular changes are not fully understood but likely reflect the interplay of several factors at the individual and societal level. The trend of increasing mental health problems, such as the one seen among young people in Norway, is a public health concern.

## Supplementary Information

Below is the link to the electronic supplementary material.

Supplementary file1 (PDF 69 KB)

Supplementary file2 (DOCX 100 KB)

## Data Availability

Data used in this study are presented in Table 1. Any additional data are available for researchers to access through the respective data owners.

## References

[1] Bor W, Dean AJ, Najman J, Hayatbakhsh R (2014) Are child and adolescent mental health problems increasing in the 21st century? A systematic review. Aust N Z J Psychiatry 48(7):606–616 24829198 10.1177/0004867414533834

[2] Collishaw S (2015) Annual research review: secular trends in child and adolescent mental health. J Child Psychol Psychiatry 56(3):370–393 25496340 10.1111/jcpp.12372

[3] Potrebny T, Wiium N, Lundegård MM-I (2017) Temporal trends in adolescents’ self-reported psychosomatic health complaints from 1980–2016: a systematic review and meta-analysis. PLoS One 12(11):e0188374 10.1371/journal.pone.0188374 PMC5705135 29182644

[4] Twenge JM (2011) Generational differences in mental health: are children and adolescents suffering more, or less? Am J Orthopsychiatry 81(4):469–472 21977931 10.1111/j.1939-0025.2011.01115.x

[5] Ask H, Handal M, Hauge LJ, Reichborn-Kjennerud T, Skurtveit S (2020) Incidence of diagnosed pediatric anxiety disorders and use of prescription drugs: a nation-wide registry study. Eur Child Adolesc Psychiatry 29(8):1063–1073 31641902 10.1007/s00787-019-01419-0

[6] Cosma A, Stevens G, Martin G, Duinhof EL, Walsh SD, Garcia-Moya I et al (2020) Cross-national time trends in adolescent mental well-being from 2002 to 2018 and the explanatory role of schoolwork pressure. J Adolesc Health 66(6, Supplement):S50–S58 10.1016/j.jadohealth.2020.02.010 PMC8131201 32446609

[7] Ottová-Jordan V, Smith ORF, Gobina I, Mazur J, Augustine L, Cavallo F et al (2015) Trends in multiple recurrent health complaints in 15-year-olds in 35 countries in Europe, North America and Israel from 1994 to 2010. Eur J Publ Health 25(suppl_2):24–27 10.1093/eurpub/ckv015 25805782

[8] Firebaugh F (2010) Analyzing data from repeated surveys. In: Marsden PV, Wright JD (eds) Handbook of survey research. Emerald Group Publishing, Leeds

[9] Bakken A (2022) Ungdata 2022 Nasjonale resultater. NOVA rapport 5/22. Report No.: 16/20. NOVA, OsloMet, Oslo

[10] Krokstad S, Weiss DA, Krokstad MA, Rangul V, Kvaløy K, Ingul JM et al (2022) Divergent decennial trends in mental health according to age reveal poorer mental health for young people: repeated cross-sectional population-based surveys from the HUNT Study, Norway. BMJ Open 12(5):e057654 35584877 10.1136/bmjopen-2021-057654 PMC9119156

[11] Potrebny T, Wiium N, Haugstvedt A, Sollesnes R, Torsheim T, Wold B et al (2019) Health complaints among adolescents in Norway: a twenty-year perspective on trends. PLoS ONE 14(1):e0210509 30625222 10.1371/journal.pone.0210509 PMC6326500

[12] Potrebny T, Wiium N, Haugstvedt A, Sollesnes R, Wold B, Thuen F (2021) Trends in the utilization of youth primary healthcare services and psychological distress. BMC Health Serv Res 21(1):115 33536017 10.1186/s12913-021-06124-w PMC7860003

[13] von Soest T, Wichstrøm L (2014) Secular trends in depressive symptoms among Norwegian adolescents from 1992 to 2010. J Abnorm Child Psychol 42(3):403–415 23888312 10.1007/s10802-013-9785-1

[14] von Soest T, Wichstrøm L (2014) Secular trends in eating problems among Norwegian adolescents from 1992 to 2010. Int J Eating Disord 47(5):448–457 10.1002/eat.22271 24610169

[15] Heiervang E, Stormark KM, Lundervold AJ, Heimann M, Goodman R, Posserud M-B et al (2007) Psychiatric disorders in Norwegian 8-to 10-year-olds: an epidemiological survey of prevalence, risk factors, and service use. J Am Acad Child Adolesc Psychiatry 46(4):438–447 17420678 10.1097/chi.0b013e31803062bf

[16] Tørmoen AJ, Myhre M, Walby FA, Grøholt B, Rossow I (2020) Change in prevalence of self-harm from 2002 to 2018 among Norwegian adolescents. Eur J Public Health 30(4):688–692 32134469 10.1093/eurpub/ckaa042 PMC7445045

[17] Bang L, Hartz I, Furu K, Odsbu I, Handal M, Suren P et al (2022) Psykiske plager og lidelser hos barn og unge 2022. Accessed 31 May 2022

[18] Kauhanen L, Wan Mohd Yunus WMA, Lempinen L, Peltonen K, Gyllenberg D, Mishina K et al (2022) A systematic review of the mental health changes of children and young people before and during the COVID-19 pandemic. Eur Child Adolesc Psychiatry 32(6):995–1013 10.1007/s00787-022-02060-0 PMC9373888 35962147

[19] von Soest T, Kozák M, Rodríguez-Cano R, Fluit DH, Cortés-García L, Ulset VS et al (2022) Adolescents’ psychosocial well-being one year after the outbreak of the COVID-19 pandemic in Norway. Nat Hum Behav 6(2):217–228 35058644 10.1038/s41562-021-01255-w

[20] Page MJ, McKenzie JE, Bossuyt PM, Boutron I, Hoffmann TC, Mulrow CD et al (2021) The PRISMA 2020 statement: an updated guideline for reporting systematic reviews. Syst Rev 10(1):89 33781348 10.1186/s13643-021-01626-4 PMC8008539

[21] Derogatis LR, Lipman RS, Rickels K, Uhlenhuth EH, Covi L (1974) The Hopkins Symptom Checklist (HSCL): a self-report symptom inventory. Behav Sci 19(1):1–15 4808738 10.1002/bs.3830190102

[22] Strand BH, Dalgard OS, Tambs K, Rognerud M (2003) Measuring the mental health status of the Norwegian population: a comparison of the instruments SCL-25, SCL-10, SCL-5 and MHI-5 (SF-36). Nord J Psychiatry 57(2):113–118 12745773 10.1080/08039480310000932

[23] Sund AM, Larsson B, Wichstrøm L (2011) Prevalence and characteristics of depressive disorders in early adolescents in central Norway. Child Adolesc Psychiatry Mental Health 5(1):1–13 10.1186/1753-2000-5-28 PMC3215923 21880127

[24] Wichstrøm L (1999) The emergence of gender difference in depressed mood during adolescence: the role of intensified gender socialization. Dev Psychol 35(1):232 9923478

[25] Inchley J, Currie D, Cosma A, Samdal O (2018) Health Behaviour in School aged Children (HBSC) Study Protocol: background, methodology and mandatory items for the 2017/18 survey.CAHRU, St. Andrews

[26] Haugland S, Wold B (2001) Subjective health complaints in adolescence—reliability and validity of survey methods. J Adolesc 24(5):611–624 11676508 10.1006/jado.2000.0393

[27] Heinz A, Sischka PE, Catunda C, Cosma A, García-Moya I, Lyyra N et al (2022) Item response theory and differential test functioning analysis of the HBSC-Symptom-Checklist across 46 countries. BMC Med Res Methodol 22(1):1–24 36175865 10.1186/s12874-022-01698-3 PMC9520881

[28] IBM Corp (2021) IBM SPSS Statistics for Windows, Version 28.0. IBM Corp, Armonk, NY

[29] R Core Team (2022) R: a language and environment for statistical computing. R Foundation for Statistical Computing, Vienna, Austria

[30] Viechtbauer W (2010) Conducting meta-analyses in R with the metafor package. J Stat Softw 36:1–48

[31] Gelman A, Hill J (2006) Data analysis using regression and multilevel/hierarchical models. Cambridge University Press, Cambridge

[32] Higgins JPT, Thompson SG, Deeks JJ, Altman DG (2003) Measuring inconsistency in meta-analyses. BMJ 327(7414):557–560 12958120 10.1136/bmj.327.7414.557 PMC192859

[33] Raudenbush SW (2009) Analyzing effect sizes: random effects models. In: Cooper H, Hedges LV, Valentine JC (eds) The handbook of research synthesis and meta-analysis, 2nd edn. Russell Sage Foundation, New York

[34] Bjertnæs AA, Fossum IN, Oma I, Bakken KS, Arne T, Holten-Andersen MN (2020) A cross-sectional study of the relationship between mental health problems and overweight and obesity in adolescents. Front Public Health 8:334 32984230 10.3389/fpubh.2020.00334 PMC7477482

[35] Kleppang AL, Thurston M, Hartz I, Hagquist C (2019) Psychological distress among Norwegian adolescents: changes between 2001 and 2009 and associations with leisure time physical activity and screen-based sedentary behaviour. Scand J Public Health 47(2):166–173 28669312 10.1177/1403494817716374

[36] Harrer M, Cuijpers P, Furukawa TA, Ebert DD (2021) Doing meta-analysis with R: a hands-on guide. Chapman & Hall/CRC Press, Boca Raton, FL and London

[37] Collishaw S, Maughan B, Natarajan L, Pickles A (2010) Trends in adolescent emotional problems in England: a comparison of two national cohorts twenty years apart. J Child Psychol Psychiatry Allied Disciplines 51(8):885–894 10.1111/j.1469-7610.2010.02252.x 20497281

[38] Tick NT, van der Ende J, Verhulst FC (2008) Ten-year trends in self-reported emotional and behavioral problems of Dutch adolescents. Soc Psychiatry Psychiatr Epidemiol 43(5):349–355 18264808 10.1007/s00127-008-0315-3

[39] Hagquist C (2010) Discrepant trends in mental health complaints among younger and older adolescents in Sweden: an analysis of WHO data 1985–2005. J Adolesc Health 46(3):258–264 20159503 10.1016/j.jadohealth.2009.07.003

[40] Mishina K, Tiiri E, Lempinen L, Sillanmäki L, Kronström K, Sourander A (2018) Time trends of Finnish adolescents’ mental health and use of alcohol and cigarettes from 1998 to 2014. Eur Child Adolesc Psychiatry 27(12):1633–1643 29704065 10.1007/s00787-018-1158-4

[41] Thorisdottir IE, Asgeirsdottir BB, Sigurvinsdottir R, Allegrante JP, Sigfusdottir ID (2017) The increase in symptoms of anxiety and depressed mood among Icelandic adolescents: time trend between 2006 and 2016. Eur J Public Health 27(5):856–861 28957485 10.1093/eurpub/ckx111

[42] De Looze M, Cosma AP, Vollebergh WA, Duinhof EL, De Roos S, van Dorsselaer S et al (2020) Trends over time in adolescent emotional wellbeing in the Netherlands, 2005–2017: links with perceived schoolwork pressure, parent-adolescent communication and bullying victimization. J Youth Adolesc 49(10):2124–2135 32705608 10.1007/s10964-020-01280-4 PMC7495988

[43] Pitchforth J, Fahy K, Ford T, Wolpert M, Viner RM, Hargreaves DS (2019) Mental health and well-being trends among children and young people in the UK, 1995–2014: analysis of repeated cross-sectional national health surveys. Psychol Med 49(8):1275–1285 30201061 10.1017/S0033291718001757 PMC6518382

[44] Keyes KM, Gary D, O’Malley PM, Hamilton A, Schulenberg J (2019) Recent increases in depressive symptoms among US adolescents: trends from 1991 to 2018. Soc Psychiatry Psychiatr Epidemiol 54(8):987–996 30929042 10.1007/s00127-019-01697-8 PMC7015269

[45] Wiens K, Bhattarai A, Pedram P, Dores A, Williams J, Bulloch A et al (2020) A growing need for youth mental health services in Canada: examining trends in youth mental health from 2011 to 2018. Epidemiol Psychiatr Sci 29:e115 32299531 10.1017/S2045796020000281 PMC7214527

[46] Forslund T, Kosidou K, Wicks S, Dalman C (2020) Trends in psychiatric diagnoses, medications and psychological therapies in a large Swedish region: a population-based study. BMC Psychiatry 20(1):328 32576173 10.1186/s12888-020-02749-z PMC7313191

[47] Mojtabai R, Olfson M, Han B (2016) National trends in the prevalence and treatment of depression in adolescents and young adults. Pediatrics 138(6):e20161878 27940701 10.1542/peds.2016-1878 PMC5127071

[48] Mojtabai R, Olfson M (2020) National trends in mental health care for US adolescents. JAMA Psychiat 77(7):703–714 10.1001/jamapsychiatry.2020.0279 PMC7097842 32211824

[49] Reas DL, Rø Ø (2018) Time trends in healthcare-detected incidence of anorexia nervosa and bulimia nervosa in the Norwegian National Patient Register (2010–2016). Int J Eat Disord 51(10):1144–1152 30265747 10.1002/eat.22949

[50] Högberg B (2021) Educational stressors and secular trends in school stress and mental health problems in adolescents. Soc Sci Med 270:113616 33348271 10.1016/j.socscimed.2020.113616

[51] Henriksen J, Nielsen PF, Bilenberg N (2012) New Danish standardization of the child behaviour checklist. Danish Med J 59(7):A4462 22759842

[52] Sellers R, Maughan B, Pickles A, Thapar A, Collishaw S (2015) Trends in parent- and teacher-rated emotional, conduct and ADHD problems and their impact in prepubertal children in Great Britain: 1999–2008. J Child Psychol Psychiatry 56(1):49–57 24953088 10.1111/jcpp.12273

[53] Twenge JM (2017) iGen: why today’s super-connected kids are growing up less rebellious, more tolerant, less happy—and completely unprepared for adulthood—and what that means for the rest of us. Simon and Schuster, New York, NY

[54] Appel M, Marker C, Gnambs T (2020) Are social media ruining our lives? a review of meta-analytic evidence. Rev Gen Psychol 24(1):60–74

[55] Orben A (2020) Teenagers, screens and social media: a narrative review of reviews and key studies. Soc Psychiatry Psychiatr Epidemiol 55(4):407–414 31925481 10.1007/s00127-019-01825-4

[56] Orben A, Przybylski AK (2019) The association between adolescent well-being and digital technology use. Nat Hum Behav 3(2):173–182 30944443 10.1038/s41562-018-0506-1

[57] Keles B, McCrae N, Grealish A (2020) A systematic review: the influence of social media on depression, anxiety and psychological distress in adolescents. Int J Adolesc Youth 25(1):79–93

[58] Braghieri L, Levy R, Makarin A (2022) Social media and mental health. Am Econ Rev 112(11):3660–3693

[59] Hjetland GJ, Finserås TR, Sivertsen B, Colman I, Hella RT, Skogen JC (2022) Focus on self-presentation on social media across sociodemographic variables, lifestyles, and personalities: a cross-sectional study. Int J Environ Res Public Health 19(17):11133 36078843 10.3390/ijerph191711133 PMC9518022

[60] Twenge JM, Haidt J, Lozano J, Cummins KM (2022) Specification curve analysis shows that social media use is linked to poor mental health, especially among girls. Acta Psychol 224:103512 10.1016/j.actpsy.2022.103512 35101738

[61] Punamäki R-L, Wallenius M, Nygård C-H, Saarni L, Rimpelä A (2007) Use of information and communication technology (ICT) and perceived health in adolescence: the role of sleeping habits and waking-time tiredness. J Adolesc 30(4):569–585 16979753 10.1016/j.adolescence.2006.07.004

[62] Hakala PT, Rimpelä AH, Saarni LA, Salminen JJ (2006) Frequent computer-related activities increase the risk of neck–shoulder and low back pain in adolescents. Eur J Public Health 16(5):536–541 16524936 10.1093/eurpub/ckl025

[63] Smith DJ, Rutter M (1995) Psychosocial disorders in young people: time trends and their causes. Academia Europaea/John Wiley, Chichester

[64] West P, Sweeting H (2003) Fifteen, female and stressed: changing patterns of psychological distress over time. J Child Psychol Psychiatry 44(3):399–411 12635969 10.1111/1469-7610.00130

[65] Boer M, Cosma A, Twenge JM, Inchley J, Jeriček Klanšček H, Stevens GWJM (2023) National-level schoolwork pressure, family structure, internet use, and obesity as drivers of time trends in adolescent psychological complaints between 2002 and 2018. J Youth Adolesc 52(10):2061–2077 37349663 10.1007/s10964-023-01800-y PMC10371956

[66] Högberg B, Strandh M, Hagquist C (2020) Gender and secular trends in adolescent mental health over 24 years—the role of school-related stress. Soc Sci Med 250:112890 32143086 10.1016/j.socscimed.2020.112890

[67] Sweeting H, West P, Young R, Der G (2010) Can we explain increases in young people’s psychological distress over time? Soc Sci Med 71(10):1819–1830 20870334 10.1016/j.socscimed.2010.08.012 PMC2981856

[68] Knapstad M, Sivertsen B, Knudsen AK, Smith ORF, Aarø LE, Lønning KJ et al (2021) Trends in self-reported psychological distress among college and university students from 2010 to 2018. Psychol Med 51(3):470–478 31779729 10.1017/S0033291719003350 PMC7958482

[69] Gagné T, Henderson C, McMunn A (2023) Is the self-reporting of mental health problems sensitive to public stigma towards mental illness? A comparison of time trends across English regions (2009–19). Soc Psychiatry Psychiatr Epidemiol 58(4):671–680 36473961 10.1007/s00127-022-02388-7 PMC9735159

[70] Jorm AF, Mackinnon AJ, Hart LM, Reavley NJ, Morgan AJ (2020) Effect of community members’ willingness to disclose a mental disorder on their psychiatric symptom scores: analysis of data from two randomised controlled trials of mental health first aid training. Epidemiol Psychiatr Sci 29:e46 10.1017/S2045796019000404 PMC8061127 31397261

