# Supplementary material for: Secular trends in mental health problems among young people in Norway: a review and meta-analysis
Source: Eur Child Adolesc Psychiatry. 2024 Feb 16;34(1):69–81. doi: 10.1007/s00787-024-02371-4 (PMC11805846; doi:10.1007/s00787-024-02371-4)
Supplement: Supplementary file 2 — Supplementary file2 (DOCX 100 KB) [file 787_2024_2371_MOESM2_ESM.docx]

**Secular trends in mental health problems among young people in Norway:**

**A review and meta-analysis.**

**Supplementary results from individual studies**

**Associations between mean symptom scores, survey year and age.**

Supplementary Table S1 shows the associations between mean symptom scores and survey year and age, for each survey separately. The Young in Norway (YiN) surveys showed a significant increase in mean symptom scores from 1992 to 2002 for both females and males, followed by a decrease between 2002-2010 for both females and males. The Young in Oslo (YiO) surveys showed a significant increase from 1996 to 2006 for females but not males. The Young-HUNT surveys showed a significant increase from 1996 to 2007 for females but not male, and a significant increase from 2007 to 2018 for both genders. The Living Conditions surveys (SILC) showed non-significant changes in all comparisons between 1998 and 2012. However, there was a significant increase from 2015 to 2019 for both females and males.

The SHoT surveys showed a significant increase for both genders from 2010 to 2014 and from 2014 to 2018. The Ungdata surveys showed a significant decrease from 2010-2013 to 2014-2016 for males, whereas no notable change was observed for females. In the period 2014-2016 to 2017-2019, both males and females showed a significant increase. The HBSC study showed a significant increase among females between 1994-2002, thereafter stable levels between 2002-2006, increase between 2006-2010, stable from 2010-2014 and increase between 2014-2018. Among females increase in age was associated with increase symptom scores. For males, there was a stable trend between 1994-2010, thereafter a decrease from 2010-2014 and finally an increase between 2014-2018.

For all individual surveys, except SHoT and SILC, a one-year increase in age was significantly associated with an increase in mean symptom scores in standardized deviation units (ranging from .041 to .128). These surveys contain adolescents between 11 and 19 years of age. In the SHoT survey, age showed a significant negative relationship with mean outcome scores for females, but a significant positive relationship for males. Of note, this survey comprises the oldest age group (18 to 24 years of age). Lastly, in SILC the relationship between age and mean outcome scores were non-significant for both genders, with the exception of the 2015 to 2019 comparison, where age was significantly associated with mean outcome scores for males, but not females.

**Supplementary Table S1.** *Linear regressions showing associations between mean symptom scores, survey year and age, by sex.*

| **Survey** | **Variables** | **Females** | | **Males** | |
| --- | --- | --- | --- | --- | --- |
|  |  | ***SMD* (SE)** | ***p*** | ***SMD*(SE)** | ***p*** |
| **YiN** | 1992-2002 | .259 (.019) | **<.001** | .400 (.019) | **<.001** |
|  | 2002-2010 | -.054 (.020) | **.008** | -.111 (.021) | **<.001** |
|  | Age | .073 (.004) | **<.001** | .041 (.004) | **<.001** |
| **Young-HUNT** | 1996-2007 | .151 (.021) | **<.001** | .029 (.022) | .179 |
|  | 2007-2018 | .432 (.021) | **<.001** | .176 (.023) | **<.001** |
|  | Age | .106 (.005) | **<.001** | .075 (.005) | **<.001** |
| **YiO** | 1996-2006 | .079 (.019) | **<.001** | .003 (.020) | .895 |
|  | Age | .128 (.011) | **<.001** | .113 (.011) | **<.001** |
| **HBSC** | 1994-1998 | .093 (.035) | **.007** | -.007 (.034) | .840 |
|  | 1998-2002 | .092 (.034) | **.008** | .052 (.035) | .131 |
|  | 2002-2006 | -.004 (.035) | .901 | .063 (.035) | .070 |
|  | 2006-2010 | .153 (.038 | **<.001** | -.049 (.037) | .180 |
|  | 2010-2014 | -.043 (.041) | .294 | -.087 (.043) | **.040** |
|  | 2014-2018 | .113 (.048) | **.018** | .204 (.050) | **<.001** |
|  | Age | .091 (.010) | **<.001** | .017 (.010) | .078 |
| **SILC** | 1998-2002 | -.104 (.072) | .149 | .017 (.077) | .822 |
|  | 2002-2005 | .135 (.079) | .085 | -.049 (.085) | .568 |
|  | 2005-2008 | .157 (.083) | .059 | .049 (.094) | .602 |
|  | 2008-2012 | .030 (.088) | .737 | .076 (.102) | .454 |
|  | 2015-2019 | .267 (.060) | **<.001** | .162 (.058) | **.006** |
|  | Age (1998 to 2012) | -.013 (.009) | .177 | .011 (.010) | .296 |
|  | Age (2015 to 2019) | .019 (.011) | .100 | .036 (.011) | **.001** |
| **SHoT** | 2010-2014 | .177 (.019) | **<.001** | .102 (.027) | **<.001** |
|  | 2014-2018 | .189 (.012) | **<.001** | .085 (.017) | **<.001** |
|  | Age | -.008 (.001) | **<.001** | .016 (.002) | **<.001** |
| **Ungdata** | (2010-2013) - (2014-2016) | .002 (.008) | .770 | -.086 (.009) | **<.001** |
|  | 2014-2016) - (2017-2019) | .136 (.010) | **<.001** | .148(.011) | **<.001** |
|  | Age | .126 (.002) | **<. 001** | .105(.003) | **<.001** |

*Note:* SMD = Standardized mean difference. SE = Standard error.

**Associations between proportions scoring above cut-off (>2) on symptom scores, survey year and age.**

Supplementary Table S2 shows the log-odds of scoring above cut-off (>2) on symptom scores by survey year and age, for each survey separately. For Young-HUNT, YiO and SHoT surveys, the proportions scoring above cut-off increased over time for both females and males. In the YiN survey, the proportion scoring above cut-off increased significantly from 1992 to 2002 but decreased significantly from 2002 to 2010. For SILC, results were mixed, and the only significant change was evident from 2015 to 2019 among females. For Ungdata, the proportion scoring above cut-off decreased for males but remained stable for females in the period 2010-2013 to 2014-2016. In the period 2014-2016 to 2017-2019, the proportion increased among males and females. For the HBSC study the proportion of females scoring over the cut-off study increased between 1994-1998 and between 2006-2010 and was stable the rest of the study period. For males, the proportion scoring over the cut-off study increased between 1998-2002 and 2014-2018 and was stable the rest of the study period. For both genders scores over the cut-off was associated with age.

Associations between proportion scoring above cut-off and age followed the exact same pattern as described in the previous section, where older youth were significantly more likely to score above cut-off. The exceptions were the SHoT survey, where younger individuals were more likely to score above cut-off; and SILC where age showed a significant relationship for males in the 2015-2019 comparison only.

**Supplementary Table S2.** *Logistic regressions showing associations between proportions scoring above cut-off (>2) on symptom scores, survey year and age, by sex.*

| **Survey** | **Variables** | **Females** | | **Males** | |
| --- | --- | --- | --- | --- | --- |
|  |  | ***Log-Odds (SE)*** | ***p*** | ***Log-Odds (SE)*** | ***p*** |
| **YiN** | 1992-2002 | .563 (.042) | **<.001** | 1.103 (.057) | **<.001** |
|  | 2002-2010 | -.106 (.044) | **.015** | -.132 (.050) | **.009** |
|  | Age | .128 (.009) | **<.001** | .078 (.012) | **<.001** |
| **Young-HUNT** | 1996-2007 | .400 (.052) | **<.001** | .104 (.073) | .151 |
|  | 2007-2018 | .739 (.049) | **<.001** | .470 (.069) | **<.001** |
|  | Age | .203 (.012) | **<.001** | .153 (.016) | **<.001** |
| **YiO** | 1996-2006 | .132 (.039) | **.001** | -.019 (.041) | .639 |
|  | Age | .253 (.022) | **<.001** | .211 (.022) | **<.001** |
| **HBSC** | 1994-1998 | .219 (.071) | **.002** | -.093 (.077) | .226 |
|  | 1998-2002 | .131 (.070) | .062 | .153 (.077) | **.046** |
|  | 2002-2006 | .059 (.072) | .407 | .104 (.075) | .168 |
|  | 2006-2010 | .202 (.077) | **.008** | .115 (.078) | .141 |
|  | 2010-2014 | -.144 (.085) | .089 | -.100 (.090) | .267 |
|  | 2014-2018 | .168 (.098) | .085 | .276 (.104) | **.008** |
|  | Age | .162 (.020) | **<.001** | .047 (.021) | **.027** |
| **SILC** | 1998-2002 | -.353 (.278) | .204 | .543 (.370) | .142 |
|  | 2002-2005 | .608 (.286) | **.033** | -.571 (.422) | .176 |
|  | 2005-2008 | .140 (.254) | .580 | -.115 (.536) | .830 |
|  | 2008-2012 | .127 (.258) | .622 | 1.077 (.491) | **.028** |
|  | 2015-2019 | .555 (.160) | **.001** | .542 (.237) | **.022** |
|  | Age (1998 to 2012) | -.018 (.031) | .572 | .064 (.049) | .190 |
|  | Age (2015 to 2019) | .054 (.030) | .077 | .139 (.046) | **.002** |
| **SHoT** | 2010-2014 | .343 (.047) | **<.001** | .271 (.085) | **.002** |
|  | 2014-2018 | .413 (.027) | **<.001** | .295 (.049) | **<.001** |
|  | Age | -.008 (.003) | **.007** | .040 (.005) | **<.001** |
| **Ungdata** | (2010-2013) - (2014-2016) | .000(.0182) | .989 | -.150(.020) | **<.001** |
|  | 2014-2016) - (2017-2019) | .245 (.0175) | **<.001** | .280(.027) | **<.001** |
|  | Age | .238(.005) | **<.001** | .196(.005) | **<.001** |

Note. SE = standard error.

**Supplementary results from meta-analysis**

To inform our modeling strategy we conducted preliminary analyses comparing the fit of a linear and non-linear functions (quadric and cubic functions) of the time trend. Based on both on the total sample and gender stratified analyses, the best fit was found for a (log) linear model (Figure S1 and Table S3).

**Supplementary Table S3.** *Fit of models comparing linear, quadratic, and cubic slopes of the time trend.*

|  | log likelihood | AIC | BIC | AICc |
| --- | --- | --- | --- | --- |
| **Total sample** |  |  |  |  |
| **Linear** | **50.59559** | **-93.19119** | **-84.31316** | **-92.55627** |
| Quadratic | 49.47472 | -88.94944 | -77.92598 | -87.96584 |
| Cubic | 48.47529 | -84.95058 | -71.81265 | -83.52685 |
| **Girls** |  |  |  |  |
| **Linear** | **50.20798** | **-92.41595** | **-86.42992** | **-90.98738** |
| Quadratic | 50.47022 | -90.94045 | -83.61177 | -88.63276 |
| Cubic | 49.34287 | -86.68574 | -78.08182 | -83.18574 |
| **Boys** |  |  |  |  |
| **Linear** | **55.31723** | **-102.63446** | **-96.64843** | **-101.20589** |
| Quadratic | 53.13046 | -96.26093 | -88.93225 | -93.95324 |
| Cubic | 53.52164 | -95.04328 | -86.43936 | -91.54328 |

*Note.* AIC: Akaike Information Criteria, BIC: Bayesian Information Criteria,

AICc: Corrected Akaike Information Criteria. Smaller values (i.e., more negative) of AIC, BIC, and AICc signifies better fitting models.

**Supplementary Figure S1.** *Predicted slopes of trends by meta-regression models.*

**A B**

*Note.* This figure shows the predicted slopes of the time trend from multilevel meta-regression analyses among girls (panel **A)** and boys (panel **B)** comparing linear, quadratic, and cubic slopes of the time trend. The points represent the estimates from the individual surveys with point sizes drawn proportional to the inverse of the standard errors such that more precise estimates yields larger points.
